# Environment-friendly wood fibre composite with high bonding strength and water resistance

**DOI:** 10.1098/rsos.172002

**Published:** 2018-04-04

**Authors:** Xiaodi Ji, Yue Dong, Tat Thang Nguyen, Xueqi Chen, Minghui Guo

**Affiliations:** Key Laboratory of Bio-Based Material Science and Technology of the Ministry of Education, Northeast Forestry University, Harbin 150040, People's Republic of China

**Keywords:** wood fibre composite, chitosan-based adhesive, bonding strength, water resistance, bonding mechanism

## Abstract

With the growing depletion of wood-based materials and concerns over emissions of formaldehyde from traditional wood fibre composites, there is a desire for environment-friendly binders. Herein, we report a green wood fibre composite with specific bonding strength and water resistance that is superior to a commercial system by using wood fibres and chitosan-based adhesives. When the mass ratio of solid content in the adhesive and absolute dry wood fibres was 3%, the bonding strength and water resistance of the wood fibre composite reached the optimal level, which was significantly improved over that of wood fibre composites without adhesive and completely met the requirements of the Chinese national standard GB/T 11718-2009. Fourier transform infrared (FTIR), X-ray photoelectron spectroscopy (XPS) and X-ray diffraction (XRD) characterizations revealed that the excellent performance of the binder might partly be due to the amide linkages and hydrogen bonding between wood fibres and the chitosan-based adhesive. We believe that this strategy could open new insights into the design of environment-friendly wood fibre composites with high bonding strength and water resistance for multifunctional applications.

## 1. Introduction

With a rapidly developing economy, the demand for wood-based materials has been increasing in China, but China is a country that does not have large forestry resources. The juxtaposition of the increasing need for wood-based materials and the short supply has caused wide concerns for the people of China. However, there are large amounts of residual forest products being produced in China [1], most of which were dispersed or combusted. Using forest residue as a raw material to produce wood fibre composites is an efficient way to meet the demand for wood composites while still preserving natural forest reserves. Common wood fibre composites in the market are prepared mainly from wood fibres and formaldehyde-containing adhesives. Formaldehyde is classified as A1 carcinogenic substance by the International Cancer Research Agency (ICRA) and formaldehyde in very high concentrations could cause cancers in the ear, nose or throat. Therefore, formaldehyde emission from traditional wood fibre composites hinders the applications of the wood fibre composite. With the depletion of the petrochemical resources, the deterioration of raw materials for synthetic resin has also been becoming an issue of concern.

Numerous wood fibre composites without the addition of an adhesive or with formaldehyde-free adhesives from natural resources have been developed in the past decades [2]. Common formaldehyde-free adhesives from natural resources are usually made including lignin [3], soya protein [4], starch [5] and wheat protein [6]. However, wood fibre composites made with some of these adhesives are costly or exhibit poor water resistance. Some adhesives are synthesized through complicated process. Wood fibre composites without adhesives usually involve complex pretreatments of wood fibres, such as alkaline pretreatment, acid pretreatment, oxidation pretreatment, enzymatic pretreatment, steam explosion and steam injection [7–10]. Like wood fibre composite made with formaldehyde-free adhesives, high cost, relatively complicated pretreatment process and poor performance limit the application of wood fibre composite without adhesive in industry.

As one of the most abundant renewable and biodegradable natural polysaccharides after cellulose in nature [11,12], chitin is a main component of crab and shrimp shells. Efficient use of chitin could solve the problem of waste and pollution due to crab and shrimp shells and could further be beneficial to sustainable development. Chitosan is derived from deacetylation of chitin and is a copolymer of β-(1,4)-linked 2-acetamido-2-deoxy-d-glucopyranoses and 2-amino-2-deoxy-d-glucopyranoses. The acetic acid solution of chitosan is viscous and homogeneous. Similar to common amino resin adhesives (e.g. urea–formaldehyde resins and melamine–formaldehyde resins), there are numerous free amino groups on the molecular chains of chitosan, which endows chitosan with bonding capacity [13–15]. Compared with other natural adhesives, chitosan exhibits excellent water resistance and antibacterial property [16–18]. Chitosan shows great potential as a multifunctional environment-friendly wood fibre composite adhesive.

Chitosan is different from common adhesives which are thermoset polymers because it is a linear polymer. Cross-linking chitosan with glutaraldehyde or epichlorohydrin could endow chitosan with a networked structure and improve its performance [19]. In previous research, we synthesized a chitosan-based adhesive by cross-linking chitosan with glutaraldehyde and investigated the influence of synthetic processes of this adhesive on the performance of wood fibre composites. The properties of this adhesive were also studied (electronic supplementary material, figure S1) [2]. However, the potential bonding mechanism and the influence of mass ratio of solid content in adhesive and absolute dry wood fibres on the performance of wood fibre composites have not yet been elucidated. Herein, we fabricated a green wood fibre composite using a chitosan-based adhesive and investigated the influence of the mass ratio of solid content in adhesive and absolute dry wood fibres on the performance of the wood fibre composite. The potential bonding mechanism and the reaction between wood fibres and adhesive were also investigated [2].

## 2. Material and methods

### 2.1. Material

Wood fibres consisting of soft wood fibres and hard wood fibres were procured from the Greater Khingan Range Hengyou Furniture Co. Ltd and were composed of cellulose (46.70 wt%), hemicelluloses (29.17 wt%) and lignin (22.39 wt%). The original moisture content of fibres was approximately 18%. Chitosan (CAS No. 9012-76-4) powder with a deacetylation of more than 95% and molecular weight from 100 000 to 150 000 Da was supplied by Shanghai Sun Chemical Technology Co. Ltd. Acetic acid (CH_3_COOH, CAS No. 64-19-7, AR) was purchased from Harbin Kaimeisi Technology Co. Ltd. Glutaraldehyde (CHO(CH_2_)_3_CHO, 50%, CAS No. 111-30-8, AR) was bought from Tianjin Ruijinte Chemical Co. Ltd. Distilled water was self-made in our laboratory. A commercial similar wood fibre composite using urea–formaldehyde resin procured from Oasis Forestry Industry Co. Ltd was used as the control group. All chemicals were used as received without any further purification.

### 2.2. Preparation of chitosan-based adhesives and wood fibre composites

In this paper, the mass ratios of solid content in adhesive and absolute dry wood fibres were 1%, 2%, 3%, 4% and 5%. Approaches for preparing wood fibre composites were the same, while the amounts of chemical reagents used were variable (table 1). When the mass ratio of solid content in adhesive and absolute dry wood fibres was 1%, the chitosan-based adhesives and wood fibre composites were synthetized as follows: chitosan powder (1 g) was added into a beaker which contained distilled water *X* (34.48 g) and stirred until the chitosan powder was dispersed uniformly in distilled water. Meanwhile, acetic acid (0.67 g) was poured into a beaker containing distilled water *Y* (34.48 g) and stirred until a homogeneous acetic acid solution was formed. Afterwards, the acetic acid solution was poured into the beaker containing chitosan and distilled water under rapid agitation with a magnetic stirrer until a homogeneous chitosan solution was formed. Simultaneously, glutaraldehyde solution (0.5 g, 50 wt%) was diluted with distilled water *Z* (34.48 g). The chitosan solution served as the main agent, while the diluted glutaraldehyde solution served as the cross-linking agent.

**Table 1.** Stoichiometries of chemical reagents used for synthesis of chitosan-based adhesives.

mass ratio^a^	chitosan (g)	acetic acid (g)	glutaraldehyde (g, 50 wt%)	distilled water *X* (g)	distilled water *Y* (g)	distilled water *Z* (g)
1	1	0.67	0.5	34.48	34.48	34.48
2	2	1.33	1	34.30	34.30	34.30
3	3	2	1.5	34.25	34.25	34.25
4	4	2.67	2	34.18	34.18	34.18
5	5	3.33	2.5	34.07	34.07	34.07

^a^Mass ratio refers to the mass ratios of solid content in the chitosan-based adhesive and absolute dry wood fibres.

**Figure 1.**
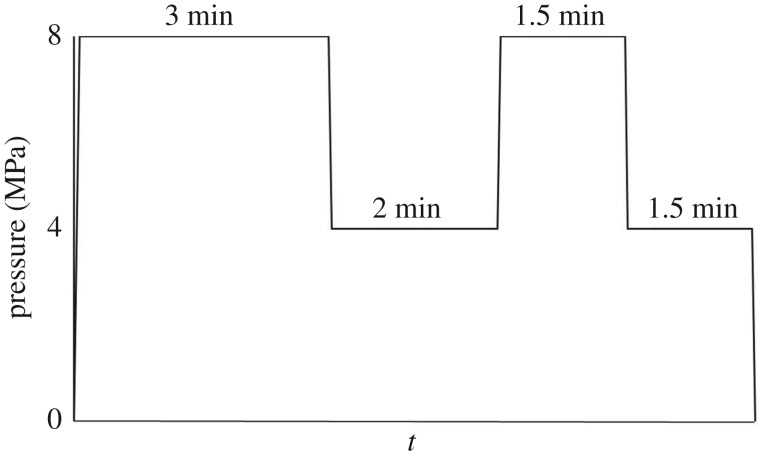
Programme schedule for hot-pressing process.

Before mixing the wood fibres and the chitosan-based adhesive, the wood fibres were dried in an oven at 80°C to reduce the moisture content from 18% to approximately 6%. Then the wood fibres were mixed in a high-speed mixer (Zhangjiagang Tongsha Plastic Machinery Co. Ltd) at 750 r.p.m. At the same time, the chitosan solution and glutaraldehyde solution were mixed up and stirred for a while. Then the adhesive was poured into the wood fibres immediately under strong agitation. Thereafter, the mixture containing wood fibres and adhesive was filled into a forming box (250 × 250 mm) and pre-pressed under 1 MPa of pressure to form a mat. Then the mat was placed between two parallel flat plates (400 × 400 mm) of a mechanically controlled oil-heated press to implement the final hot (170°C) pressing according to a pre-programmed hot-pressing schedule (figure 1). A 5 mm steel gauge was used to control the thickness of wood fibre composite. Finally, the edges of wood fibre composite were cut off by 30 mm using a table saw, resulting in a 220 × 220 × 5 mm board with a target density of 0.8 ± 0.02 g cm^−3^. The wood fibre composite was conditioned at a relative humidity of 40% and room temperature for 2 days before bonding strength and water resistance tests. Wood fibre composites with mass ratios of solid content in adhesive and absolute dry wood fibres of 1%, 2%, 3%, 4% and 5% were designated as WFC 1, WFC 2, WFC 3, WFC 4 and WFC 5, respectively. Wood fibre composites without adhesive and the control group were designated as WFC 0 and CG, respectively.

### 2.3. Bonding strength and water resistance tests

The bonding strength and water resistance of wood fibre composite were evaluated through mechanical and dimensional properties (modulus of rupture, MOR; modulus of elasticity, MOE; internal bond strength, IB; thickness swell, TS and water adsorption, WA), which were measured according to the Chinese national standard GB/T 17657-2013. The MOR represents the rate of the bending moment and flexing modulus under the highest load, while the MOE represents the rate of the stress and strain of the load within the range of the elastic limit. The IB represents the rate of the highest damage tension perpendicular to the surface of the specimen and the specimen surface area. The TS and WA refer to the increase in the thickness and weight of a specimen soaked in water for 24 h at room temperature. The MOR and the MOE of the composites were determined by executing three-point static bending tests on specimens with a size of 200 × 50 mm at a cross-head speed of 5 mm min^−1^. The IB was determined by pulling the specimen (50 × 50 mm) apart in a perpendicular direction. The specimens were sanded to take away the low-density surface area. Thereafter, metal fixtures were adhered on each side of the specimen using hot melting adhesive. A tensile load with a cross-head speed of 0.5 mm min^−1^ was applied on each metal fixture until failing happened in the specimen. The TS and WA were determined by measuring the thickness and weight of specimens with a size of 50 × 50 mm before and immediately after the 24 h immersing process. The MOR and MOE measurements were conducted on 12 repetitions, while IB, TS and WA measurements were conducted on eight repetitions.

### 2.4. Characterization of wood fibre composite

Scanning electron microscopy (SEM) images of the fractured wood fibre composites after IB tests were taken with a microscope (Quanta200, FEI, USA) operated at 12.5 kV. To make the samples conductive, sample surfaces were coated with a thin layer of gold using an ion sputter coater prior to the SEM analysis. FTIR spectra of wood fibres and wood fibre composites were collected using Nicolet Magna-IR560 E.S.P. with the ATR technique. The spectra were recorded at a resolution of 4 cm^−1^ by accumulating 32 scans. Surface chemistry of wood fibres and wood fibre composite was characterized by X-ray photoelectron spectroscopy (XPS) on a VG Thermo probe ESCA system (Thermo Electron, USA). The chemical shifts relative to C1 (284.3 eV) for C2, C3 and C4 were 1.5 ± 0.1 eV, 3 ± 0.1 eV and 4 ± 0.1 eV, respectively. X-ray diffraction (XRD) patterns were obtained using an X-ray diffractometer (D/max 2200, Rigaku, Japan) and the relative crystallinity (CI) was calculated according to Segal method [20] as follows:

2.1 $$\text{CI}=\frac{I_{002}-I_{\mathrm{am}}}{I_{002}}\times 100\%,$$

where *I*_002_ represents the maximum intensity of both crystalline and amorphous region of the material and *I*_am_ represents the intensity of the amorphous region of the material.

## 3. Results and discussion

### 3.1. Bonding strength and water resistance

**Figure 2.**
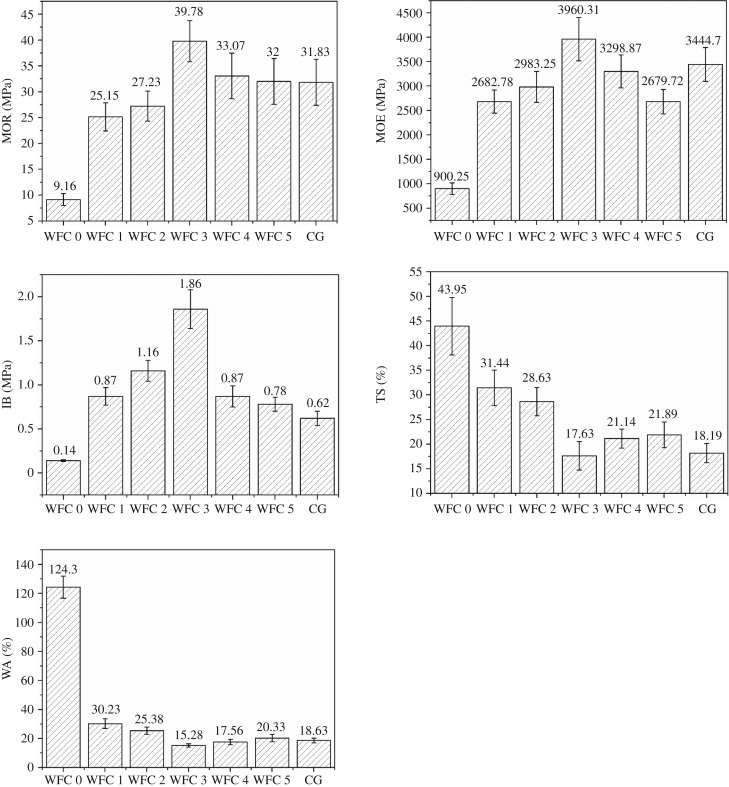
Influence of adhesive amount on the wood fibre composites performance.

The influence of the mass ratio of solid content in adhesive and absolute dry wood fibres on the performance of the wood fibre composites is shown in figure 2. It is worth noting that the trends for MOR, MOE and IB can be divided into two stages. During the first stage, there is a correlation to the mass ratio in the range from 0% to 3% and the MOR, MOE and IB increased from 9.16 MPa, 900.25 MPa and 0.14 MPa to 39.78 MPa, 3960.31 MPa and 1.86 MPa, respectively. The increase in bonding strength was remarkable and indicates that there is an optimal mass ratio of solid content in the adhesive and absolute dry wood fibres for the mechanical performance of the wood fibre composites.

During the second stage corresponding to the mass ratio in the range from 3% to 5%, the mechanical performance of wood fibre composites deteriorated with increasing mass ratio. The change in bonding strength was reflected in a decline in MOR, MOE and IB from 39.78 MPa, 3960.31 MPa and 1.86 MPa to 32 MPa, 2679.72 MPa and 0.78 MPa, respectively. These results show that the mechanical performance of the wood fibre composite cannot increase with increasing mass ratio and will reach a limit at the mass ratio of 3%. As mass ratio increased from 3% to 5%, the adhesive solution concentration also increased. Thus, the chitosan content and glutaraldehyde content in the adhesive solution increased, which led to an adhesive solution with increasing viscosity and non-uniform distribution of wood fibres and adhesive [21]. The high chitosan content and glutaraldehyde content in the adhesive solution can also cause premature curing of the adhesive [22]. The combination of non-uniform distribution of wood fibres and adhesive and premature curing of the adhesive were responsible for the deterioration of mechanical performance of wood fibre composites when the mass ratio was more than 3%.

The change trends of water resistance of wood fibre composites were similar to those of mechanical performance and can also be divided into two stages. The water resistance increased during the first stage correlating to mass ratio in the range from 0 to 3% and then declined in the second stage corresponding to the mass ratio in the range from 3% to 5% as reflected in the first increase and then decrease in TS and WA. The optimal TS and WA were 17.63% and 15.28%, respectively, both of which decreased significantly compared with that of WFC 0. The improvement of water resistance was partly attributed to the strong adhesion between wood fibres which were adhered closely to each other. The other reason was that chitosan shows good water resistance as it can prevent water from entering its molecular chains [16]. While the subsequent decline of water resistance was also ascribed to the non-uniform distribution of wood fibres and adhesive and premature curing of the adhesive, both of which caused the decrease in adhesion among wood fibres.

In summary, the MOR, MOE, IB, TS and WA all reached an optimal level when the mass ratio of solid content in adhesive and absolute dry wood fibres was 3%. The optimal mechanical performance and water resistance both improved significantly compared to WFC 0 and met the requirements for the Chinese national standard. WFC 3 bears favourable comparison with CG in terms of performance and even shows much better IB than CG. These results demonstrate that an environment-friendly wood fibre composite with high bonding strength and water resistance could be fabricated through the appropriate addition of this chitosan-based adhesive.

### 3.2. Fourier transform infrared analysis

**Figure 3.**
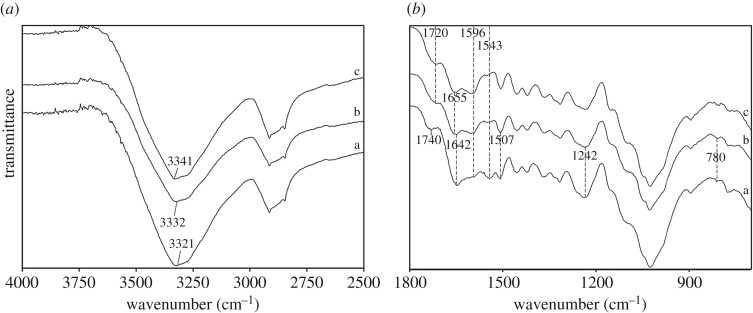
FTIR spectra of (*a*) wood fibres, (*b*) WFC 0 and (*c*) WFC 3.

Figure 3 shows the FTIR spectra of wood fibres, WFC 0 and WFC 3. Compared to wood fibres, some changes were observed in the FTIR spectrum of WFC 0. The peaks at 1642 cm^−1^ and 1543 cm^−1^ corresponding to ester linkages decreased after hot pressing [22]. The ester linkages are components of hemicelluloses and extractives. Therefore, this decline shows that extractives and hemicelluloses were chemically changed during the hot-pressing process. The peaks at 1507 cm^−1^, 1242 cm^−1^ and 780 cm^−1^, which have been reported to be associated with C=C symmetric stretching vibration, C–O stretching vibration and CH out-of-plane vibrations corresponding to lignin, respectively, weakened after the hot-pressing process [23,24]. These results indicate that the lignin was degraded. The peak at 1596 cm^−1^, which belongs to superposition of C=C linkages of lignin and C=O linkages, strengthened after hot pressing [23,24], and this change implies the generation of some low molecular weight organics including furan, furfural and methyl-furfural due to the degradation of hemicelluloses, lignin and extractives. The formation of these low molecular weight organics resulted in the improvement of C=O linkages and was effective in the self-bonding of WFC 0 [25,26]. The peak at 1740 cm^−1^ in the FTIR spectrum of wood fibres corresponds to the association of COOH of hemicelluloses, while the FTIR spectrum of WFC 0 shows a peak at 1720 cm^−1^ corresponding to C=O linkages. The peak at 1720 cm^−1^ in the FTIR spectrum of WFC 0 was weaker than the peak at 1740 cm^−1^ in the spectrum of wood fibres [23]. The decline of this peak indicates degradation of hemicellulose and the shift can be attributed to the generation of hydrogen bonds associated with COOH groups. The peak corresponding to OH groups in the FTIR spectrum of wood fibres was at 3321 cm^−1^, but it moved to 3332 cm^−1^ in the spectrum of WFC 0. The shift is due to the appearance of hydrogen bonds related to OH groups. Therefore, some hydrogen bonds between COOH groups and OH groups of wood fibres occurred after hot pressing. In summary, the newly formed low molecular weight organics and hydrogen bonds resulted in self-bonding of wood fibres and thus were responsible for the performance of WFC 0 [27].

The mechanical properties and water resistance of WFC 0 were quite poor, while those of WFC 3 were excellent. This difference implies that the performance of WFC 3 relied on the self-bonding of wood fibres and adhesion between wood fibres caused by the chitosan-based adhesive. Therefore, elucidation of reactions between wood fibres and the chitosan-based adhesive can help to elucidate the bonding mechanism.

In comparison with WFC 0, the peak related to amide linkages at 1655 cm^−1^ strengthened in the FTIR spectrum of WFC 3 and this change implies a reaction between the amine groups of chitosan-based adhesive and carbonyl groups from wood fibres, which resulted in the formation of amide linkages [28]. The peak at 1596 cm^−1^ also strengthened in the FTIR spectrum of WFC 3. This peak was the superposition of the C=C linkages of lignin and the C=O amide linkages. This change is also a proof of amide linkages. The peaks associated with OH groups at 3332 cm^−1^ in the FTIR spectrum of WFC 0 further shifted to higher wavenumbers at 3341 cm^−1^ in the FTIR spectrum of WFC 3 and this change indicates more hydrogen bonds between OH groups of wood fibres occurred [29]. These hydrogen bonds may exist between OH groups of wood fibres and OH and amine groups of the chitosan-based adhesive. The peak at 1720 cm^−1^ corresponding to C=O in the FTIR spectrum of WFC 3 was stronger than that of WFC 0, which was attributed to glutaraldehyde existing in the chitosan-based adhesives.

The above FTIR analysis shows the formation of amide linkages and hydrogen bonds between wood fibres and the chitosan-based adhesives, which was essential for the excellent performance of WFC 3.

### 3.3. X-ray photoelectron spectroscopy analysis

The XPS analysis was used to identify the surface chemistry of the wood fibres, WFC 0 and WFC 3. It was observed that the O/C ratio of wood fibres was 0.28, while the O/C ratio of WFC 0 increased to 0.33 after the hot-pressing process (table 2). Combined with the results of FTIR analysis, it was inferred that the improvement of O/C ratio after hot pressing was attributed to the newly formed low molecular weight organics which are rich in oxygen-containing groups. The O/C ratio of WFC 3 further rose to 0.34 due to the addition of the chitosan-based adhesive, which contains an abundance of oxygen-containing groups [1]. The emerging N in WFC 3 was also due to the addition of the chitosan-based adhesive.

**Table 2.** Surface chemistry component of wood fibres, WFC 0 and WFC 3 determined by XPS.

	elements (%)			carbon components C1s (%)				binding energy (eV)				
sample	C	O	N	C1	C2	C3	C4	C1	C2	C3	C4	O/C ratio
wood fibres	78.1	21.9	0	71.3	20.9	4.7	3.1	284.3	285.8	287.3	288.3	0.28
WFC 0	75.2	24.8	0	59.1	32.7	5.2	3.0	284.3	285.8	287.2	288.4	0.33
WFC 3	71.1	24.4	4.5	57.2	34.0	5.3	3.5	284.3	285.8	287.3	288.3	0.34

**Figure 4.**
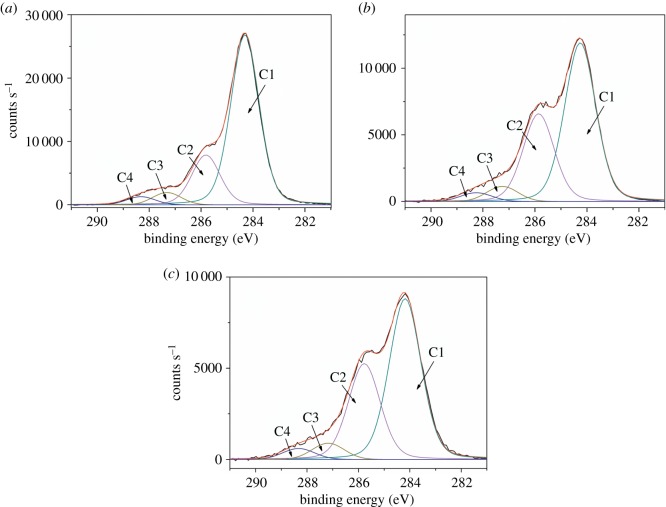
High-resolution XPS spectra of (*a*) wood fibres, (*b*) WFC 0 and (*c*) WFC 3.

Figure 4 shows the C 1 s spectra of wood fibres, WFC 0 and WFC 3 with high-resolution scans, which were deconvoluted into four components for all specimens: C1 (C–C/C–H/C=C) at 284.3 ± 0.1 eV, C2 (C–O/C–N) at 285.8 ± 0.1 eV, C3 (C=O/O–C–O) at 287.3 ± 0.1 eV and C4 (O–C=O/N–C=O) at 288.3 ± 0.1 eV [15,30]. Compared to wood fibres, C1 of WFC 0 decreased, while C2 and C3 increased. C1 only exists in the lignin and extractives, and thus the decrease of C1 was partly due to the degradation of lignin and extractives during pressing. The other reason was the relative increase of C2 and C3 due to the generation of oxygen-containing low molecular weight organics, such as furan, furfural and methyl-furfural. C4 of WFC 0 declined slightly compared to that of wood fibres and C4 only exists in hemicelluloses. This phenomenon is indicative of hemicellulose degradation. These results are consistent with the FTIR analysis.

By comparison with WFC 0, the C1 of WFC 3 decreased and C2 and C3 increased due to the addition of the chitosan-based adhesive. C4 of WFC 0 exhibited a downward trend due to the degradation of hemicelluloses, while C4 of WFC 3 went up. There were no C4 components in the chitosan-based adhesive itself (electronic supplementary material, figure S1). Therefore, the increase in C4 could be attributed to the newly generated amide linkages between wood fibres and the chitosan-based adhesives, which was beneficial for the composite properties of WFC 3 and aligns with FTIR analysis.

### 3.4. X-ray diffraction analysis

**Figure 5.**
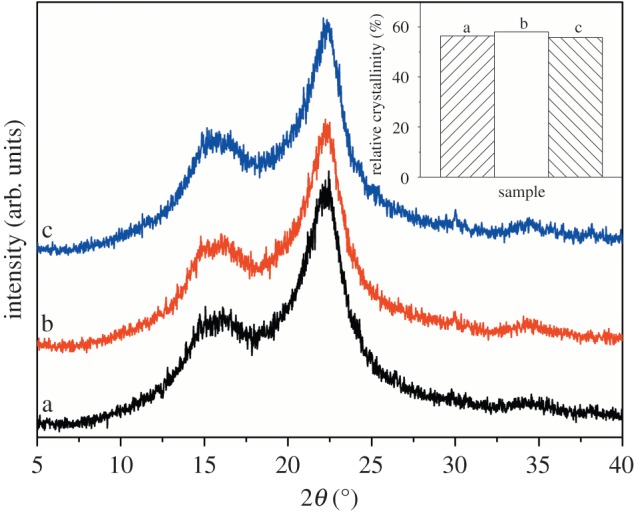
XRD patterns obtained for (*a*) wood fibres, (*b*) WFC 0 and (*c*) WFC 3.

Figure 5 shows the XRD diffraction patterns and the relative crystallinity of the wood fibres, WFC 0 and WFC 3. The crystal structure of cellulose in the wood fibres, WFC 0 and WFC 3 was similar and corresponded to the typical cellulose I pattern because both the diffraction peaks at 2*θ* = 16.5° and 22.6° could be observed in all three diffraction profiles [31]. Therefore, the hot pressing and addition of chitosan-based adhesive did not change the crystal structure. However, the relative crystallinity of these samples differed from each other. Crystallinity is a major factor that influences the mechanical and dimensional properties of wood-based materials. The influence of hot pressing, adhesives and other components of wood fibres on the crystallization behaviour and supermolecular structure of cellulose could be reflected through calculating the crystallinity. Compared with wood fibres, WFC 0 showed slightly increased crystallinity. The wood fibres were compacted closely and densely during the hot-pressing process, and hence the possibility of generation of hydrogen bonds between OH groups of cellulose increased, which caused the improvement of relative crystallinity [32]. The self-bonding of WFC 0 partly relied on the hydrogen bonds due to pressure-driven fibril aggregation [33]. The degradation of hemicelluloses, lignin and extractives resulted in a relative increase in cellulose content, which could contribute to the relative increase in crystallinity.

Different from the increase in the relative crystallinity of WFC 0 compared to wood fibres, the relative crystallinity of WFC 3 declined slightly compared to WFC 0, which is partly attributed to the reduction of the possibility of hydrogen bonds between hydroxyls of cellulose caused by the amide linkages and hydrogen bonds between amino and hydroxyl groups of chitosan and hydroxyl groups of cellulose. The decrease in crystallinity might also be associated with the disruption of the surface of the crystalline regions by the cured chitosan-based adhesives [34], which agrees well with the earlier findings by other researchers [35]. In addition, the chitosan-based adhesive is amorphous [2]. Hence, the amorphous structure of the chitosan-based adhesive incorporated into cellulose also led to a reduction in crystallinity [36].

The XRD analysis agrees with the FTIR and XPS analyses and a combination of these results shows that the bonding mechanism of WFC 3 might be dependent on the amide linkages and hydrogen bonds between wood fibres and the chitosan-based adhesive.

### 3.5. Scanning electron microscopy image analysis

**Figure 6.**
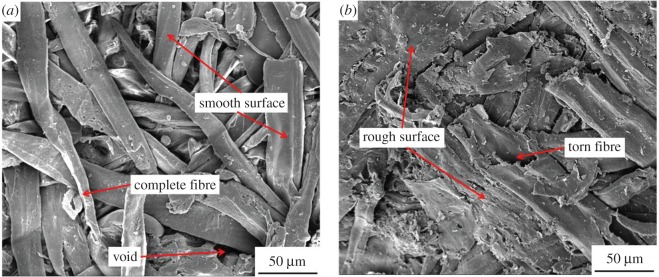
SEM image of the fractured surfaces of (*a*) WFC 0 and (*b*) WFC 3.

Figure 6 shows the fractured surfaces of WFC 0 and WFC 3 after IB tests. The original circular cell cavities of wood fibres were compacted during the hot-pressing process. But there were no signs of deformed cell walls, which indicates that wood fibre structures are maintained. The wood fibres in WFC 0 were randomly distributed and interlaced with one another and made it easy to form interlocks among individual wood fibres. The wood fibres in WFC 0 originally contacted closely to each other due to the hot-pressing process. However, as shown in figure 6, wood fibres were loosely contacted and some voids existed in the fracture surface of WFC 0. A pulling force divided WFC 0 into two parts and some wood fibres were pulled out directly from WFC 0 during IB testing. Some deformations of wood fibres due to the hot pressing were recovered after IB tests. These phenomena indicate that the adhesion between wood fibres was poor and did not prevent deformation recovery and wood fibres from pulling out.

The wood fibre surfaces of WFC 3 were rougher than those of WFC 0 due to the uniform distribution of chitosan-based adhesive among the wood fibre surfaces. The wood fibres in WFC 3 were more compacted than those in WFC 0 and exhibited fewer voids. Moreover, there was even a torn fibre in WFC 3, which was pulled apart during the IB test. The torn fibre was snapped rather than pulled out completely, indicating strong adhesion among the wood fibres, which prevented wood fibres from pulling out and deformation recovery. Therefore, it can be concluded that the adhesion among wood fibres in WFC 3 was strong and ensured excellent mechanical performance and water resistance of WFC 3.

**Figure 7.**
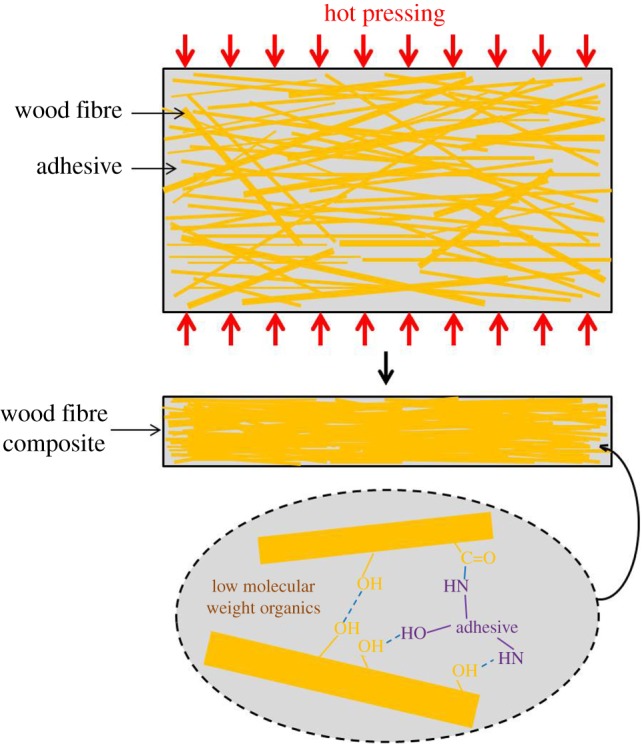
Illustrations of the preparation process and bonding mechanism for the wood fibre composite.

In summary, from the results of FTIR spectroscopy, XPS analysis, XRD patterns, SEM images and the previous works of ours and other researchers, the partial potential bonding mechanism of these wood fibre composites could be conjectured roughly (figure 7). Firstly, wood fibres became closely and tightly interlocked under high pressure. Then hemicellulose, lignin and extractives in the wood fibres degraded and low molecular weight organics were generated during hot pressing. The further use of high pressure during hot pressing allowed for the cellulose, hemicelluloses, lignin and extractives to be pressed close to each other. The combination of high pressure and temperature allowed for the generation of hydrogen bonds among the different polymers. Meanwhile, amide linkages were formed between carbonyl groups of wood fibres and the amine groups of the chitosan-based adhesive. Hydrogen bonds between wood fibres and chitosan-based fibres were also generated. A combination of these interlocks, amide linkages, hydrogen bonds and low molecular weight organics contributed to the excellent performances of wood fibre composite. The whole bonding mechanism is very complicated and it is extremely hard to fully clarify all the bonding mechanisms in this paper. The bonding mechanism given above is only a part of the whole bonding mechanism and is of guiding significance and lays foundation for fabrication of the wood fibre composite and further research. There is still a lot of work to do with the bonding mechanism.

## 4. Conclusion

In conclusion, allowing for the shortage of wood-based materials and the negative effects of formaldehyde emissions in traditional wood fibre composites and inspired by chitosan, a simple approach was used that could fabricate environment-friendly wood fibre composites with high bonding strength and water resistance by using a chitosan-based adhesive and residual forest wood fibres. The obtained wood fibre composite with a mass ratio of solid content in adhesive and absolute dry wood fibres of 3% exhibited excellent MOR, MOE, IB, TS and WA up to 39.78 MPa, 3960.31 MPa, 1.86 MPa, 17.63% and 15.28%, respectively. These results were superior to the wood fibre composite without an adhesive and a commercially available product. Furthermore, the potential bonding mechanism analysis shows that the excellent performance of the chitosan-based adhesive was partly reliant on a combination of wood fibres interlocks, newly formed amide linkages, hydrogen bonds and low molecular weight organics. Because chitosan also shows outstanding antibacterial properties, the proposed method provides a simple strategy for designing and developing multifunctional environment-friendly wood fibre composite with excellent performance. Further research will continue to systematically investigate the bonding mechanisms and versatility of this wood fibre composite.

## Supplementary Material

Structures of chitosan-based adhesive

## Data accessibility

Part of the datasets are provided in the main text and the others are uploaded as part of the electronic supplementary material.

## References

[1] Liu F, Yu R, Guo M 2016 Hydrothermal carbonization of forestry residues: influence of reaction temperature on holocellulose-derived hydrochar properties. J. Mater. Sci. 52, 1–11. (doi:10.1007/s10853-016-0465-8)

[2] Ji X, Li B, Yuan B, Guo M 2017 Preparation and characterizations of a chitosan-based medium-density fiberboard adhesive with high bonding strength and water resistance. Carbohyd. Polym. 176, 273–280. (doi:10.1016/j.carbpol.2017.08.100) 10.1016/j.carbpol.2017.08.100 28927608

[3] Ji X, Guo M 2018 Preparation and properties of a chitosan-lignin wood adhesive. Int. J. Adhes. Adhes. 82, 8–13. (doi:10.1016/j.ijadhadh.2017.12.005)

[4] Luo J, Luo J, Li X, Li K, Gao Q, Li J 2016 Toughening improvement to a soybean meal-based bioadhesive using an interpenetrating acrylic emulsion network. J. Mater. Sci. 51, 1–12. (doi:10.1007/s10853-016-0180-5)

[5] Nordqvist P, Khabbaz F, Malmström E 2010 Comparing bond strength and water resistance of alkali-modified soy protein isolate and wheat gluten adhesives. Int. J. Adhes. Adhes. 30, 72–79. (doi:10.1016/j.ijadhadh.2009.09.002)

[6] Bai X, Wang G, Gong C, Yu Y, Liu W, Wang D 2017 Co-pelletizing characteristics of torrefied wheat straw with peanut shell. Bioresour. Technol. 233, 373–381. (doi:10.1016/j.biortech.2017.02.091) 28285230 10.1016/j.biortech.2017.02.091

[7] Ariawan D, Mohd Ishak ZA, Salim MS, Mat Taib R, Ahmad Thirmizir MZ 2017 Wettability and interfacial characterization of alkaline treated kenaf fiber-unsaturated polyester composites fabricated by resin transfer molding. Polym. Composite 38, 507–515. (doi:10.1002/pc.23609)

[8] Tao X, Li J, Zhang P, Nabi M, Jin S, Li F, Wang S, Ye J 2017 Reinforced acid-pretreatment of *Triarrhena lutarioriparia* to accelerate its enzymatic hydrolysis. Int. J. Hydrogen Energ. 42, 18 301–18 308. (doi:10.1016/j.ijhydene.2017.04.149)

[9] Ryan CC, Bardosova M, Pemble ME 2017 Structural and mechanical properties of a range of chitosan-based hybrid networks loaded with colloidal silica and polystyrene particles. J. Mater. Sci. 52, 8338–8347. (doi:10.1007/s10853-017-1051-4)

[10] Zhang D, Zhang A, Xue L 2015 A review of preparation of binderless fiberboards and its self-bonding mechanism. Wood. Sci. Technol. 49, 661–679. (doi:10.1007/s00226-015-0728-6)

[11] Vnučec D, Žigon J, Mikuljan M, Kamke FA, Šernek M, Kutnar A, Goršek A 2017 Bonding of densified beech wood using adhesives based on thermally modified soy proteins. Euro. J. Wood Wood Prod. 75, 767–776. (doi:10.1007/s00107-017-1164-0)

[12] Takahashi I, Sugimoto T, Takasu Y, Yamasaki M, Sasaki Y, Kikata Y 2011 Bamboo fiber reinforced thermoplastic molding made of steamed wood flour. J. Mater. Sci. 46, 6841–6849. (doi:10.1007/s10853-011-5644-z)

[13] Jiang Y, Liu B, Xu J, Pan K, Hou H, Hu J, Yang J 2018 Cross-linked chitosan/β-cyclodextrin composite for selective removal of methyl orange: adsorption performance and mechanism. Carbohyd. Polym. 182, 106–114. (doi:10.1016/j.carbpol.2017.10.097) 10.1016/j.carbpol.2017.10.097 29279104

[14] Kim U-J, Lee YR, Kang TH, Choi JW, Kimura S, Wada M 2017 Protein adsorption of dialdehyde cellulose-crosslinked chitosan with high amino group contents. Carbohyd. Polym. 163, 34–42. (doi:10.1016/j.carbpol.2017.01.052) 10.1016/j.carbpol.2017.01.052 28267516

[15] Hopper AP, Dugan JM, Gill AA, Fox OJ, May PW, Haycock JW, Claeyssens F 2014 Amine functionalized nanodiamond promotes cellular adhesion, proliferation and neurite outgrowth. Biomed. Mater. 9, 045 009–045 011. (doi:10.1088/1748-6041/9/4/045009) 10.1088/1748-6041/9/4/045009 25029630

[16] Mima S, Miya M, Iwamoto R, Yoshikawa S 2010 Highly deacetylated chitosan and its properties. J. Appl. Polym. Sci. 28, 1909–1917. (doi:10.1002/app.1983.070280607)

[17] Shahbazi M, Rajabzadeh G, Ahmadi SJ 2017 Characterization of nanocomposite film based on chitosan intercalated in clay platelets by electron beam irradiation. Carbohyd. Polym. 157, 226–235. (doi:10.1016/j.carbpol.2016.09.018) 10.1016/j.carbpol.2016.09.018 27987922

[18] Zhu L, Dai J, Chen L, Chen J, Na H, Zhu J 2017 Design and fabrication of imidazolium ion-immobilized electrospun polyurethane membranes with antibacterial activity. J. Mater. Sci. 52, 2473–2483. (doi:10.1007/s10853-016-0542-z)

[19] Xu Y, Han J, Lin H 2017 Fabrication and characterization of a self-crosslinking chitosan hydrogel under mild conditions without the use of strong bases. Carbohyd. Polym. 156, 372–379. (doi:10.1016/j.carbpol.2016.09.046) 10.1016/j.carbpol.2016.09.046 27842836

[20] Ji X, Dong Y, Yuan B, Li B, Guo M 2018 Influence of glutaraldehyde on the performance of a lignosulfonate/chitosan-based medium density fiberboard adhesive. J. Appl. Polym. Sci. 135, 45 870–45 878. (doi:10.1002/app.45870)

[21] Segal LC, Creely J, Martin AEJ, Conrad CM 1959 An empirical method for estimating the degree of crystallinity of native cellulose using the X-ray diffractometer. Text. Res. J. 29, 786–794. (doi:10.1177/004051755902901003)

[22] Roberts GAF, Taylor KE 1989 Chitosan gels, 3. The formation of gels by reaction of chitosan with glutaraldehyde. Macromol. Chem. Phys. 190, 951–960. (doi:10.1002/macp.1989.021900504)

[23] Essabir H, Bensalah MO, Rodrigue D, Bouhfid R, Qaiss AK 2016 Biocomposites based on Argan nut shell and a polymer matrix: effect of filler content and coupling agent. Carbohyd. Polym. 143, 70–83. (doi:10.1016/j.carbpol.2016.02.002) 10.1016/j.carbpol.2016.02.002 27083345

[24] Dai D, Fan M 2015 Preparation of bio-composite from wood sawdust and gypsum. Ind. Crop. Prod. 74, 417–424. (doi:10.1016/j.indcrop.2015.05.036)

[25] Rowell RM, McSweeny JD 2008 Heat treatments of wood fibers for self-bonding and stabilized fiberboards. Mol. Cryst. Liq. Cryst. 483, 307–325. (doi:10.1080/15421400801918179)

[26] Okuda N, Hori K, Sato M 2006 Chemical changes of kenaf core binderless boards during hot pressing (I): influence of the pressing temperature condition. J. Wood Sci. 52, 244–245. (doi:10.1007/s10086-005-0761-4)

[27] Xia G, Sadanand V, Ashok B, Reddy KO, Zhang J, Rajulu AV 2015 Preparation and properties of cellulose/waste leather buff biocomposites. Int. J. Polym. Anal. Ch. 20, 693–703. (doi:10.1080/1023666X.2015.1081132)

[28] Chen SH et al. 2013 Synthesis and characterization of reinforced poly(ethylene glycol)/chitosan hydrogel as wound dressing materials. Macromol. Mater. Eng. 298, 429–438. (doi:10.1002/mame.201200054)

[29] Wang J, Wei L, Ma Y, Li K, Li M, Yu Y, Wang L, Qiu H 2013 Collagen/cellulose hydrogel beads reconstituted from ionic liquid solution for Cu(II) adsorption. Carbohyd. Polym. 98, 736–743. (doi:10.1016/j.carbpol.2013.06.001) 10.1016/j.carbpol.2013.06.001 23987406

[30] Rück-Braun K, Petersen MÅ, Michalik F, Hebert A, Przyrembel D, Weber C, Ahmed SA, Kowarik S, Weinelt M 2013 Formation of carboxy- and amide-terminated alkyl monolayers on Silicon(111) investigated by ATR-FTIR, XPS, and X-ray scattering: construction of photoswitchable surfaces. Langmuir 29, 11 758–11 769. (doi:10.1021/la402068d) 10.1021/la402068d 23971741

[31] Yin J, Yuan T, Lu Y, Song K, Li H, Zhao G, Yin Y 2017 Effect of compression combined with steam treatment on the porosity, chemical compositon and cellulose crystalline structure of wood cell walls. Carbohyd. Polym. 155, 163–172. (doi:10.1016/j.carbpol.2016.08.013) 10.1016/j.carbpol.2016.08.013 27702500

[32] Hu JP, Guo MH 2015 Influence of ammonium lignosulfonate on the mechanical and dimensional properties of wood fiber biocomposites reinforced with polylactic acid. Ind. Crop. Prod. 78, 48–57. (doi:10.1016/j.indcrop.2015.09.075)

[33] Nilsson H, Galland S, Larsson PT, Gamstedt EK, Nishino T, Berglund LA, Iversen T 2010 A non-solvent approach for high-stiffness all-cellulose biocomposites based on pure wood cellulose. Composites. Sci. Technol. 70, 1704–1712. (doi:10.1016/j.compscitech.2010.06.016)

[34] So S, Teh JW, Rudin A, Tchir WJ, Fyfe CA 2010 Effects of resole phenol adhesives on the crystallinity of cellulose. J. Appl. Polym. Sci. 39, 531–538. (doi:10.1002/app.1990.070390304)

[35] Earl WL, Vanderhart DL 1981 Observations by high-resolution carbon-13 nuclear magnetic resonance of cellulose I related to morphology and crystal structure. Macromolecule 14, 570–574. (doi:10.1021/ma50004a023)

[36] Younesi-Kordkheili H, Kazemi-Najafi S, Eshkiki RB, Pizzi A 2015 Improving urea formaldehyde resin properties by glyoxalated soda bagasse lignin. Euro. J. Wood Wood Prod. 73, 77–85. (doi:10.1007/s00107-014-0850-4)

