# Supplementary material for: Environment-friendly wood fibre composite with high bonding strength and water resistance
Source: R Soc Open Sci. 2018 Apr 4;5(4):172002. doi: 10.1098/rsos.172002 (PMC5936918; doi:10.1098/rsos.172002)
Supplement: Structures of chitosan-based adhesive [file rsos172002supp1.docx]

**Supplementary Information**

**Journal:** Royal Society Open Science

**Title:** Environmental-friendly wood fibre composite with high bonding strength and water resistance

**Authors:**

Xiaodi Ji^1^, Yue Dong^1^, Tat Thang Nguyen ^1^, Xueqi Chen^1^ and Minghui Guo^1*^

**Affiliations:**

^1^Key Laboratory of Bio-Based Material Science and Technology of the Ministry of Education, Northeast Forestry University, Harbin 150040, China

*** Corresponding author:**

Minghui Guo; E-mail:gmh1964@126.com

**Table of contents:**

Figure S1. Structures of chitosan, glutaraldehyde, self-polymerized glutaraldehyde, chitosan-based adhesive 2

Synthesis mechanism and structure of the chitosan-based adhesive 2


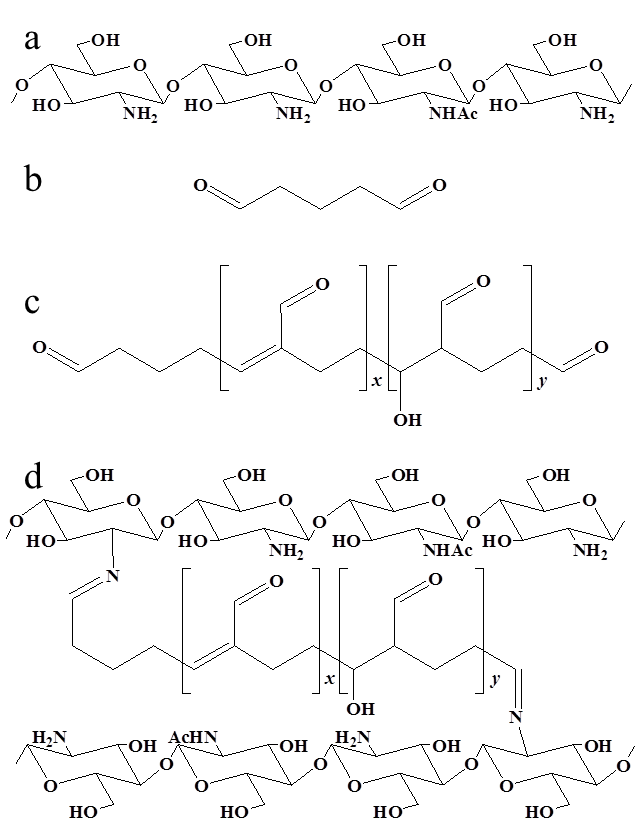


**Figure S1.** Structures of (a) chitosan, (b) glutaraldehyde monomer, (c) self-polymerized glutaraldehyde, (d) chitosan-based adhesive.

**Synthesis mechanism and structure of the chitosan-based adhesive.** When the glutaraldehyde was prepared, self-polymerization of glutaraldehyde occurred by aldol condensation. Then the self-polymerized glutaraldehyde solution was poured into the beaker which containing chitosan solution and the mixture was blended for a while. During hot pressing process, the chitosan was crosslinked with the self-polymerized glutaraldehyde through the imine linkages and thus the network structure formed. Meanwhile, wood fibres were bonded together through the chitosan-based adhesive.
